# Harmonization of epidemiological studies on dementia in Latin America Why does it matter?

**DOI:** 10.1590/1980-57642018dn13-040001

**Published:** 2019

**Authors:** Cleusa P. Ferri, Deborah Oliveira

**Affiliations:** 1 PhD, MD. Universidade Federal de São Paulo (UNIFESP), Escola Paulista de Medicina (EPM), Department of Psychiatry, São Paulo, SP, Brazil.; 2 Heath Technology Assessment Unit, Hospital Alemão Oswaldo Cruz, São Paulo, SP, Brazil.; 2 PhD, RN. Universidade Federal de São Paulo (UNIFESP), Escola Paulista de Medicina (EPM), Department of Psychiatry. São Paulo, SP, Brazil.

**Keywords:** epidemiology, harmonization, dementia, public health, methodology, epidemiologia, harmonização, demência, saúde pública, metodologia

## Abstract

Evidence on dementia in Latin America (LA) is limited and varies between and within countries, contributing to a delay in the establishment and implementation of dementia action plans by governments and services. The harmonization of standardised measurement outcomes and the use of unified databases that address the key issues affecting the LA population can help address this issue. This paper is based on a presentation delivered at a satellite Alzheimer’s Association International Conference held in April 2019, in Brazil, and aims to discuss the challenges and benefits of harmonizing epidemiological studies on dementia in the region. First, we mention some of the characteristics of LA in relation to geography, population, socioeconomic and epidemiological conditions, which could potentially affect preventative measures and dementia diagnosis in the region. Second, we cite some studies to demonstrate how research on dementia in LA is limited and uses diverse methodology. We proceed by justifying the need for harmonization of epidemiological studies in LA and discuss what type of data could be harmonised. We conclude by briefly mentioning harmonization in relation to risk factors for dementia.

## RESUMO

As evidências científicas sobre demência na América Latina (AL) são limitadas e variam entre e dentro dos países, o que contribui para um atraso no estabelecimento e na implementação de planos de ação para demência por governos e serviços. A harmonização de medidas por instrumentos padronizados e o uso de bancos de dados unificados que abordam os principais problemas que afetam a população da AL podem ajudar a resolver esse problema. Este artigo é baseado em uma apresentação realizada durante a *Alzheimer’s Association International Conference* que aconteceu em abril de 2019 no Brasil e tem como objetivo discutir os desafios e benefícios da harmonização de estudos epidemiológicos sobre demência nesta região. Primeiramente, mencionamos algumas das características da AL em relação à geografia, população, condições socioeconômicas e epidemiológicas, que podem potencialmente afetar ações de prevenção e diagnóstico de demência na região. Depois, citamos alguns estudos para demonstrar como os estudos sobre a demência na AL são limitados e utilizam metodologias diversas. Em seguida, argumentamos pela necessidade de harmonização dos estudos epidemiológicos sobre demência na AL e discutimos os tipos de dados podem ser harmonizados. Por fim, mencionamos brevemente a importância da harmonização em estudos envolvendo os fatores de risco para demência.

The global action plan in the public response to dementia (2017-2025) by the World Health Organization has provided a framework for joint efforts tackling dementia worldwide.^1^ Its seven proposed actions aim to prevent dementia, as well as to treat, care for and support people affected by the disease and their families, so they can live meaningful and dignified lives. The majority of people with dementia currently live in low- and middle-income countries,^2^ characteristics common to most nations in Latin America (LA).^3^ It is estimated that the number of people with dementia in LA will rise from 3.4 million in 2010 to 15.9 million in 2050, a 370% increase.^4^

The LA region is marked by huge ethnic, cultural, educational and socioeconomic diversity, with high levels of deprivation and income inequality in most countries. The limited provision of resources for dementia prevention, treatment and care hinders the implementation of public health actions such as those proposed by the WHO.^1^ High levels of stigma and poor professional training affect how well people with dementia can live in the region.^5^ In addition, current evidence on dementia in LA is limited and inconsistent between and within countries, delaying the establishment of goals and implementation of dementia action plans by governments and services. It is vital that research takes into account this diversity so that more accurate estimates can be produced and effective interventions designed and implemented, taking into account the specific needs of the region.^5^ One way of doing this is by encouraging the establishment of consensus through the creation of standardised measurement outcomes and unified databases that address the key issues affecting the LA population.^5^ This paper aims to discuss the challenges and benefits of harmonizing epidemiological studies on dementia in Latin America. The content of this manuscript was presented at the Satellite Symposium of the Alzheimer’s Disease International Conference held in São Paulo, Brazil, in April 2019.

## THE LATIN-AMERICAN CONTEXT

The LA region is formed by the entire continent of South America, in addition to Central America, Mexico, and some of the Caribbean countries. Current estimates indicate that the population of LA is about 650 million people.^3^ Half of this population lives in Brazil and Mexico. Brazil is not just the most populous country, but it is also the largest by geographical area.^3^

Latin America’s cultural and ethnic origins derive from the interaction of indigenous people with European settlers and immigrants, as well as with African migrants through slavery. Given the long history of colonialism, the overwhelming majority of Latin Americans speak Portuguese or Spanish. LA also has a long history of political conflicts and contradictions with a high degree of inequality, poverty and volatile economic growth. Despite some positive outcomes from poverty alleviation programmes, there is still a lot that needs to be done in terms of offering equal opportunities for all Latin Americans. Despite these continuing problems, people in LA are living longer and healthier lives with significant advances in the region regarding mortality and life expectancy. 

There are more than 50 million people over 65 in LA, with major variation in the proportion of older people between countries.^3^ According to the United Nations’ 2017 revision of the world population prospectus, the proportion of people above 65 in the region was 8% and ranged from 5% in Guatemala to 15% in Cuba and Uruguay, which reflects the same rate estimated for the United States.^3^ These variations also occur within large countries like Brazil where the populations of the south and southeast live longer than those in the north.^6^ With respect to life expectancy, this is above 70 years of age in most LA countries, again varying between countries, from 69 in Bolivia to over 80 in Cuba, for example.^3^

These differences are reflected in the ranking and contribution of different conditions to the health of the populations of different countries. When looking at the shift between 1990 and 2010 in the main causes of years of healthy life lost, or DALYS (Disability-Adjusted Life Years), we can see that countries are at different stages of the epidemiological transition and the accompanying shift from infectious diseases to chronic diseases.^7^ In general, across LA, the leading causes are chronic conditions, violence and road injury, with an increase in DALYS caused by all these factors over the past years. Countries like Bolivia, with a lower life expectancy, are at a relatively early stage of the demographic and epidemiological transition. Although lower respiratory tract infections, diarrhoea-related diseases and pre-term birth complications remain the leading causes of years of healthy life lost, their contribution to the burden of disease has decreased since 1990. Conversely, the percentage of DALYs attributable to non-communicable diseases has since increased.^7^ At the other end of the spectrum is Uruguay, with nearly 20% of its population aged over 60 and where chronic conditions remained the leading causes of years of healthy life lost between 1990 and 2010.^7^ Even though there was an overall decrease in DALYs due to these conditions in this country, Alzheimer’s disease contributed to a greater proportion of the number of healthy life years lost.

## DEMENTIA IN LATIN AMERICA: LIMITED EVIDENCE HOLDS BACK PROGRESS IN THE AREA

It is estimated that nearly 4 million Latin Americans have dementia today, representing about 7.1% of the population aged 65 and over.^8^ This figure is based on a systematic review of eight epidemiological studies on dementia prevalence conducted in six countries only, with estimates ranging from 2% in a small town in São Paulo state in Brazil, to 13.1% in Caracas, Venezuela.^8^ Data from the 10/66 studies in LA using the DSM-IV criteria have shown prevalence rates ranging from 2.5% in Caracas to 6.4% in Cuba.^9^ Data from the 10/66 studies conducted in urban areas show prevalence rates ranging from 6.5% in Peru to 11.7% in the Dominican Republic.^9^ Other studies conducted in Brazil have shown higher prevalence rates than most studies in LA (e.g. Lopes et al. found a prevalence of 12.5%;^10^ Bottino et al. reported a prevalence of 12.9%;^11^ while Cesar et al. found a prevalence of 17.5%^12^). However, most of these studies lack representativeness, where nearly all epidemiological studies on dementia in Brazil have been conducted in São Paulo state, the wealthiest in the country. 

Based on the pooled estimates of the 10/66 diagnoses, there are about 5 million people with dementia in LA today. However, if the 10/66 DSM-IV criteria algorithm is used, this figure is only 2.2 million. If the 7.1% estimate of the systematic review by Nitrini et al. is taken into account, there are 3.7 million people with dementia in LA.^8^ Significant differences in estimates, of just over two million to just under four million, may significantly affect other predictions in terms of societal burden of the disease, costs, and care needs. These figures also have important implications for patient advocacy (e.g. through non-profit organizations) and for organizing and planning health care provision. These data demonstrate the limitations and inconsistencies of the data available on dementia prevalence, but these problems are even greater in relation to incidence and mortality estimates. 

## HARMONIZATION: WHY DOES IT MATTER, AND WHAT DATA SHOULD BE HARMONISED?

With regards to dementia research, we do not argue that all studies be designed or conducted in exactly the same way, but that there should at least be consensus on good practice for conducting population-based studies on dementia (prevalence, incidence and mortality), and agreement about the best way to identify/diagnose people with dementia. 

There are several issues related to the various epidemiological studies conducted in LA. Most importantly, studies should; a) be of high overall quality; b) investigate the representativeness of populations with adequate sample sizes; and c) be conducted properly to minimize the introduction of potential biases. The decision regarding whether to conduct one- or two-phase design studies is difficult, as there is a need to consider not only the best methodological approach, but also the associated costs. The one-phase design may have advantages when incidence, risk factor and mortality studies are planned, as all participants are assessed at baseline; however, costs can limit the depth of the individual assessment performed in a one-phase design or the number of participants in a full assessment in a two-phase design. One-phase designs usually rely on a diagnosis being made using an algorithm, while two-phase studies are more likely to use a clinical gold standard assessment.

It is likely that estimates may be less or more accurate depending on the methods used in the various studies. For example, taking studies conducted in Venezuela by Maestre et al. in 2002^13^ and the 10/66 group in 2010^9^ as examples, we note that while the 10/66 one-phase design study using a DSM-IV algorithm estimated a prevalence of 2.5% in Caracas; the other study, which also used a one-phase design, only with a DSM-IV clinical diagnosis, identified a prevalence of 13.1% in Maracaibo. Is it likely that dementia prevalence in Maracaibo was more than 5 times higher than in Caracas? Some of this difference may be real, but it is probable that this disparity is partly to do with the study design and partly to do with the way dementia was diagnosed. A two-phase study depends on the sensitivity and specificity of the screening assessment used in phase one. Unfortunately, we still do not have a screening instrument that is sufficiently accurate to make the choice of a two-phase design the default choice. Psychometric tests developed in high-income countries should be adequately translated, culturally adapted and validated before being used in LA, as this can help save time and resources, as well as enable comparisons between data from LA and other countries around the world. 

There is some debate about the best criteria for dementia diagnosis. The most used criteria in epidemiological studies is the DSM-IV in its several versions. However, there are other proposed criteria that claim to be cross-cultural and more accurate for populations with low levels of literacy, such as those used in the 10/66 study. The 10/66 studies have made a huge contribution to the understanding of dementia in LA and other LMICs and have the advantage of being harmonised, adopting the same diagnostic criteria across six LA countries. Another important contribution of 10/66 is that they are conducting, or have already conducted, new prevalence studies in the same catchment areas as the one conducted in 2000, which will allow them to show changes over more than a decade in different parts of LA. What we need to be aware of with large consortiums like 10/66, or any other harmonised epidemiological study, is the potential of having a systematic error in identifying people with dementia in all these countries. How well our epidemiological studies can accurately identify people with dementia will not only affect our estimates of burden, cost, needs and care provision, but also the estimated contribution of the different dementia risk and protective factors in the region.

## A NOTE ON RISK FACTORS FOR DEMENTIA

With regard to the measuring of modifiable risk factors for dementia, it is important that we use the best method for each factor, harmonized across studies; and that the study design is robust and includes long-term follow-up. Besides doing this well, we also need to study how these factors may potentially work together in the different countries’ populations. Older people are likely to have various comorbidities, and so we need to use sophisticated approaches to understand the contribution of each factor, which are likely to vary between populations.

## CONCLUSION

In conclusion, LA is a widely diverse region and researchers need to take this diversity into account when conducting epidemiological studies; however, we do need to ensure some level of harmonization and agreement on what is considered to be best practice in epidemiological studies on dementia. This is necessary not only to make fair cross-cultural comparisons, but also to produce estimates of the burden of dementia, including its costs, that are as accurate as possible, and to organize the provision of care to meet the health needs of the population. Harmonised data can provide LA with more context-based outcomes, on which long-term care plans can be based that are affordable and sustainable, as well as being equitable and accessible to all people with dementia and their carers.

There is an urgent need to improve the quantity and quality of evidence on dementia in LA more broadly. As previously mentioned, there are many more studies on dementia prevalence in LA than on incidence and mortality, and even fewer on economic assessment. There is a need for a balance in the amount of research in each of these key areas, along with an increase in the amount of research on health services. It should be remembered that there are also many geographical areas that have not been covered at all. This is likely related to not only the overall lack of investment in research and training of researchers, but also to the lack of priority given to dementia. We also need to monitor estimates over time, allowing adjustments to the provision of care and to policies for prevention and health promotion, as well as enabling better estimates to inform care and policy development for future generations.

## References

[1] World Health Organization (2017). Global action plan on the public health response to dementia 2017 -
2025.

[2] Alzheimer's Disease International (2018). World Alzheimer Report 2018: The State of the art of dementia research:
New frontiers.

[3] United Nations (2017). World Population Prospects: Key findings & advance tables.

[4] ADI/Bupa report (2013). Dementia in the Americas: Current and future cost and prevalence of
Alzheimer's disease and other dementias.

[5] Parra MA, Baez S, Allegri R, Nitrini R, Lopera F, Slachevsky A (2018). Dementia in Latin America: Assessing the present and en-visioning
the future. Neurology.

[6] Brazilian Institute of Geography and Statistics (2019). Population projections.

[7] Institute for Health Metrics and Evaluation (IHME) Global Burden of Disease.

[8] Nitrini R, Bottino CM, Albala C, Custodio Capuñay NS, Ketzoian C, Llibre Rodriguez JJ (2009). Prevalence of dementia in Latin America: a collaborative study of
population-based cohorts. Int Psychogeriatr.

[9] Ferri CP, Prince M (2010). 10/66 Dementia Research Group: recently published survey data for
seven Latin America sites. Int Psycho-geriatr.

[10] Lopes MA, Ferrioli E, Nakano EY, Litvoc J, Bottino CMC (2012). High prevalence of dementia in a community-based survey of older
people from Brazil: association with intellectual activity rather than
education. J Alzheimers Dis.

[11] Bottino CM, Azevedo D, Tatsch M, Hototian SR, Moscoso MA, Folquitto J (2008). Estimate of dementia prevalence in a com-munity sample from São
Paulo, Brazil. Dement Geriatr Cogn Disord.

[12] César KG, Brucki SM, Takada LT, Nascimento LF, Gomes CM, Almeida MC (2016). Prevalence of Cognitive Impairment Without Dementia and Dementia
in Tremembe, Brazil. Alzheimer Dis Assoc Disord.

[13] Maestre GE, Pino-Ramírez G, Molero AE, Silva ER, Zambrano R, Falque L (2002). The Maracaibo Aging Study: population and methodological
issues. Neuroepidemiology.

